# Alteration of Microbiome Profile by D-Allulose in Amelioration of High-Fat-Diet-Induced Obesity in Mice

**DOI:** 10.3390/nu12020352

**Published:** 2020-01-29

**Authors:** Youngji Han, Haryung Park, Bo-Ra Choi, Yosep Ji, Eun-Young Kwon, Myung-Sook Choi

**Affiliations:** 1 Department of Food Science and Nutrition, Kyungpook National University, 1370 San-Kyuk Dong Puk-Ku, Daegu 41566, Korea; youngji.kor.han@gmail.com (Y.H.); borachoi15@naver.com (B.-R.C.); eykwon@knu.ac.kr (E.-Y.K.); 2 Center for Food and Nutritional Genomics Research, Kyungpook National University, 1370 San-Kyuk Dong Puk-Ku, Daegu 41566, Korea; 3 Department of Advanced Green Energy and Environment, Handong Global University, Gyeongbuk, Pohang 37554, Korea; hrpark@microbes.bio (H.P.); jiyosep@gmail.com (Y.J.)

**Keywords:** D-allulose, obesity, metagenomics, microbiome, sugar substitute

## Abstract

Recently, there has been a global shift in diet towards an increased intake of energy-dense foods that are high in sugars. D-allulose has received attention as a sugar substitute and has been reported as one of the anti-obesity food components; however, its correlation with the intestinal microbial community is not yet completely understood. Thirty-six C57BL/6J mice were divided in to four dietary groups and fed a normal diet (ND), a high-fat diet (HFD, 20% fat, 1% cholesterol, w/w), and a HFD with 5% erythritol (ERY) and D-allulose (ALL) supplement for 16 weeks. A pair-feeding approach was used so that all groups receiving the high-fat diet would have the same calorie intake. As a result, body weight and body fat mass in the ALL group were significantly decreased toward the level of the normal group with a simultaneous decrease in plasma leptin and resistin. Fecal short-chain fatty acid (SCFA) production analysis revealed that ALL induced elevated total SCFA production compared to the other groups. Also, ALL supplement induced the change in the microbial community that could be responsible for improving the obesity based on 16S rRNA gene sequence analysis, and ALL significantly increased the energy expenditure in Day(6a.m to 6pm). Taken together, our findings suggest that 5% dietary ALL led to an improvement in HFD-induced obesity by altering the microbiome community.

## 1. Introduction

Obesity is a serious global health issue that, in combination with other risk factors, is increasing the prevalence of metabolic diseases [1,2,3,4,5]. Besides fat, sugar is one of the dietary factors responsible for obesity in modern society [4,5]. Among the different types of sugar, high fructose intake is particularly problematic because, unlike glucose, fructose does not stimulate insulin secretion from pancreatic β-cells or circulating leptin levels and fails to stimulate satiety signaling [6,7,8]. Persistent dysregulation of food intake and energy homeostasis due to reduced insulin and leptin signals can accelerate energy intake, weight gain, and obesity [9].

Numerous studies targeting the prevention and treatment of obesity have incorporated prebiotics, probiotics, or synbiotics as co-adjuvants [10,11,12,13,14]. The basis for this approach is the experimental evidence showing that modifying the gut microbiome in rodents ameliorates insulin sensitivity and decreases body weight and fat mass [12,13,14]. Furthermore, there is mounting evidence that the gut microbiota plays an essential role in energy harvesting and host metabolism, suggesting a link between the gut microbiota composition and metabolic diseases, such as obesity and type 2 diabetes [12,15,16]. The production of short-chain fatty acids (SCFAs) through non-digestible carbohydrates is one of the ways in which the microbiome regulates the energy expenditure (EE) and metabolism within the host [17,18]. These findings support the idea that prebiotics, probiotics, or synbiotics may ameliorate obesity through the modulation of the gut microbiome.

D-allulose, a C-3 epimer of D-fructose, is a sugar substitute, which has 70% of the sweetness of sucrose but almost zero calories and is rarely found in nature [19]. It is only present in small quantities in commercial mixtures of d-glucose and D-fructose obtained from the hydrolysis of sucrose or the isomerization of d-glucose. Several studies have provided preliminary evidence on the impact of D-allulose on lipid metabolism in animal and human models [20,21,22]. However, the mechanism underlying the microbial action of D-allulose is still not clear. In our past study with a diet-induced obesity (DIO) mouse model, D-allulose supplementation suppressed lipid absorption in the small intestine and increased the fecal lipid contents [22]. Thus, we assumed that D-allulose would improve DIO by altering the gut microbiome profile due to the gut bacteria and diet interactions, and then we performed an animal feeding study and evaluated both the biochemical composition of the microbiome by differentially abundant genera [23,24,25].

## 2. Methods

### 2.1. Animals and Diets

A total of 40 male C57BL/6J mice (4 weeks old) were purchased from the Jackson Laboratory (Bar Harbor, ME, USA). The animals were maintained in a room with a controlled temperature (20–23 °C) and lighting (12/12 h light–dark cycle) and fed a pelletized commercial non-purified diet for 1 week after arrival. The mice were then randomly divided into four groups (*n* = 9) and fed the respective experimental diets for 16 weeks, as shown in Table 1: normal diet control (ND, American Institute of Nutrition AIN-76 semi-synthetic diet); high-fat diet control (HFD, 20% fat plus 1% cholesterol based on the AIN-76 diet); 5% erythritol (ERY, 5% erythritol substituted for sucrose in HFD, w/w), and 5% D-allulose (ALL, 5% D-allulose substituted for sucrose in HFD, w/w). D-allulose was purchased from Sigma–Aldrich (St. Louis, MO, USA). The HFD was formulated to provide 39.5% of the total energy from fat, by replacing carbohydrate energy with lard and corn oil, and had the same amounts of vitamins and minerals per kilojoule as the ND. The ALL group was fed the D-allulose diet. The HFD and ERY groups were fed iso-caloric diets based on the energy intake of the ALL group in a pair-fed manner. The mice had free access to distilled water during the experimental period. Food intake was recorded daily and body weight was monitored every 2 weeks. The SYRCLE’s risk of bias was performed as described in the Supplementary Table S1. All animal procedures were approved by the Ethics Committee for Animal Studies at Kyungpook National University, Daegu, Republic of Korea (Approval No. KNU-2016-130).

### 2.2. Plasma Lipid Profile Analysis

The plasma-free fatty acid, apolipoprotein A-I (ApoA-1), and apolipoprotein B (ApoB) levels were measured using a Nittobo enzymatic kit (Nittobo Medical Co., Tokyo, Japan). The plasma HDL-cholesterol (HDL-C), triglyceride (TG), and total cholesterol (total-C) levels were determined using commercially available enzymatic kits (Asan, Seoul, South Korea).

### 2.3. Plasma Adipokines Measurement

Plasma leptin, resistin, and adiponectin were determined using a multiplex detection kit from Bio-Rad (Hercules, CA, USA). All of the samples were assayed in duplicate and analyzed using a Luminex® 200 LabMAP™ system (Luminex, Austin, TX, USA). The data analyses were performed using the Bio-Plex Manager software version 4.1.1 (Bio-Rad).

### 2.4. EE and Whole-Body Oxygen Consumption

EE was measured using an indirect calorimeter (Oxylet; Panlab, Cornella, Spain). The mice were placed into individual metabolic chambers at 25 °C, with free access to food and water. O_2_ and CO_2_ analyzers were calibrated with highly purified gas standards. Whole-body oxygen consumption (Vo_2_) and carbon dioxide production (Vco_2_) were recorded at 3-min intervals using a computer-assisted data acquisition program (Chart 5.2; AD Instruments, Sydney, Australia) over a 24-h period. The data were averaged for each mouse. EE was calculated as follows: EE (kcal/day/ [kg of body weight × 0.75]) = Vo_2_ × 1.44 × [3.815 + (1.232 × Vo_2_/Vco_2_)].

### 2.5. Histopathology Analysis

Liver and epididymal white adipose tissue (eWAT) were removed from mice and fixed in a buffer solution of 10% formalin. All fixed tissues were processed routinely for paraffin embedding. Sections of 4 mm in thickness were prepared and stained with hematoxylin–eosin and Masson’s trichrome (MT). The stained areas were viewed under an optical microscope (Nikon, Tokyo, Japan) with 200× magnification.

### 2.6. SCFA Analysis

All SCFAs were extracted from 50 mg of mice fecal sample in 500 μL of extraction buffer (0.1 M oxalic acid, 40 mM NaN_3_) with 0.3 g of zirconium beads. After bead-beating the sample for 3 min, the samples were incubated in a shaking incubator at room temperature for 1 h and centrifuged at 16,000× *g* at 25 °C for 5 min. according to Schwiertz et al. [25]. The supernatant of the centrifuged samples was collected and transferred to a transparent gas chromatography vial for analysis using a Shimadzu GC2010 (Agilent, Santa Clara, CA, USA) equipped with a flame ionization detector (FID) and HP INNOWax column (30 m × 32 mm). The operating conditions were as follows: column heated from 100 to 180 °C at the rate of 25 °C/min; splitter temperature 260 °C; FID temperature, 260 °C; pressure, 27.1 psi.

### 2.7. Microbiota Analysis

Fecal DNA was extracted using the QIAamp DNA Stool Mini Kit (Qiagen, Hilden, Germany). Briefly, 50 mg of feces was mixed vigorously with zircon/silica beads (BioSpec Products, Oklahoma, USA) for 3 min, and the remaining protocol proceeded as described in the manufacturer’s instructions. Microbial community profiling by 16S ribosomal RNA amplicon sequencing of 5 ng/μL of fecal DNA was performed using primers specifically targeting the 16S rRNA V3/V4 region. The PCR products were purified by PCR clean-up, according to the Illumina (San Diego, CA, USA) 16S Metagenomic Sequencing Library Preparation protocol. Dual indices were attached to the samples by using the Nextera XT Index Kit (FC-131-1002), and the indexed samples were sequenced on an Illumina MiSeq system. The raw data were visualized and analyzed for beta diversity, alpha diversity, and taxonomy using QIIME [26].

### 2.8. Statistical Analysis

All phenotype data are presented as the mean ± SE or SD. Statistical analysis was performed using SPSS software version 11.0 (SPSS, Inc., Chicago, IL, USA). The statistical differences between the ND and HFD results were determined by the Student’s *t*-test. One-way ANOVA was performed to compare the HFD groups, and Turkey’s multiple-range test was performed when significant differences were identified between the groups (*p* < 0.05).

## 3. Results

### 3.1. Anti-obesity Effects of D-Allulose Supplement in DIO Mice

**Figure 1:**
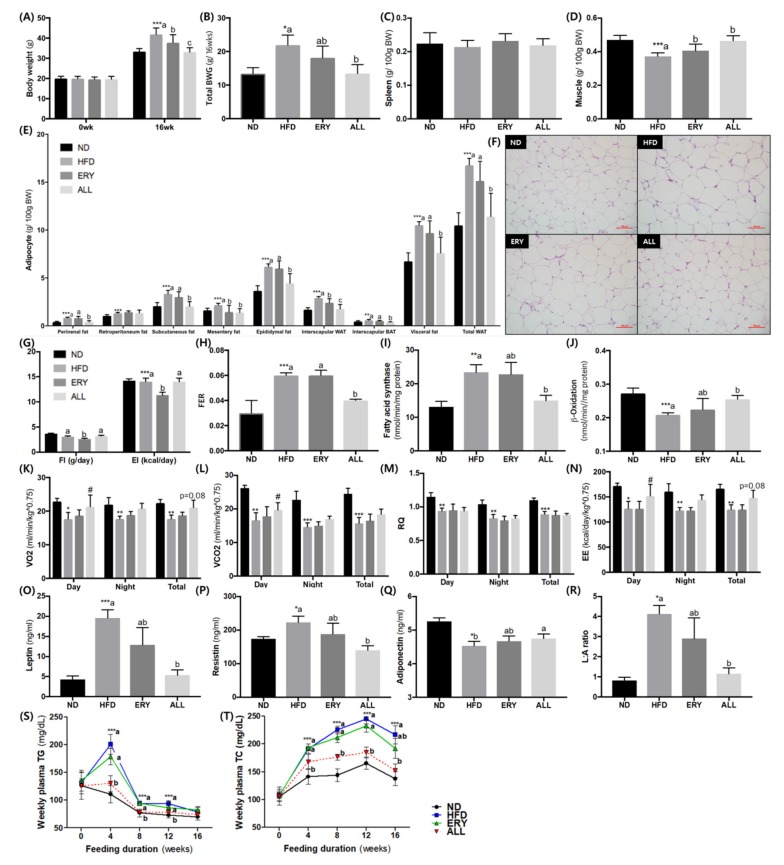
Effects of D-allulose supplementation for 16 weeks on; (**A**) Body weight; (**B**) Body weight gain; (**C**,**D**) Spleen weight and muscle weight; (**E**,**F**) Adipocyte weight and morphology; (**G**) Food intake and energy intake; (**H**) Food efficiency ratio; (**I**,**J**) Adipocyte enzyme activity-related lipid metabolism; (**K**–**N**) Metabolic rate, (**O**–**R**) adipokine levels and weekly plasma (**S**) TG and (**T**) TC. Data are mean ± SE; ND, normal diet (AIN-76); HFD, high-fat diet (AIN-76, 20% fat, 1% cholesterol); ALL, (HFD + 5% D-allulose). Mean values are significantly different for ND vs HFD, *** *p* < 0.001; Mean values are significantly different for HFD vs ALL, ^##^ *p* < 0.01, ^###^ *p* < 0.001. (B) BWG, body weight gain; (C) FI, food intake; EI, energy intake; (D) FER, food efficiency ratio = body weight gain/food intake; (E) WAT, white adipose tissue; BAT, brown adipose tissue; (F) hematoxylin and eosin (H&E)-stained transverse section of epididymal fat and liver; Representative photomicrographs of the liver are shown at ×200 magnification; (G) FI, food intake; EI, energy intake; (K) VO_2_, oxygen consumption, (L), VCO_2_, carbon dioxide production, (M) RQ, respiratory quotient, (N)EE, energy expenditure, (R) L:A ratio, Leptin:adiponectin ratio, (S) TG, triglyceride, (T) TC, total cholesterol.

At the end of the experimental period, HFD-fed mice were drastically increased in body weight relative to the ND group (Figure 1A). However, the ALL-fed animals had a lower body weight than the HFD and ERY groups due to the suppression of the total body weight gain (Figure 1A,B), which was similar among the animals fed HFD, ERY, and ND. Muscle weight increased after D-allulose supplementation for 16 weeks, whereas the spleen weight was comparable before and after the ALL diet (Figure 1C,D). In the comparison of fat mass, all types of adipocyte tissue in the HFD group weighed significantly more than in the ND group (Figure 1E) and, except for mesenteric fat, were dramatically lower in the ALL group than HFD group. These results are consistent with the epididymal morphology (Figure 1F).

The food intake and energy intake were significantly lower in the ERY group than the HFD and ALL groups, while the food efficiency ratio in the ALL group was significantly lower compared with the HFD and ERY groups (Figure 1G,H). Regarding the plasma lipid profiles among the groups of animals, the HFD group presented markedly elevated total-C, HDL-C, and non-HDL-C. In contrast, these three variables, as well as ApoA-1, were significantly decreased in the ALL group (Table 2). In eWAT, the activity of fatty acid synthase and β-oxidation activity was significantly decreased and increased, respectively, in the ALL group (Figure 1J). The metabolic rate measurements of VO_2_, VCO_2_, and EE per day were significantly increased in the ALL group relative to the HFD group (Figure 1K–N). While the adiponectin level was significantly higher in the ALL group, the leptin and resistin levels and the leptin–adiponectin ratio were significantly decreased compared with the HFD group.

**Figure 2:**
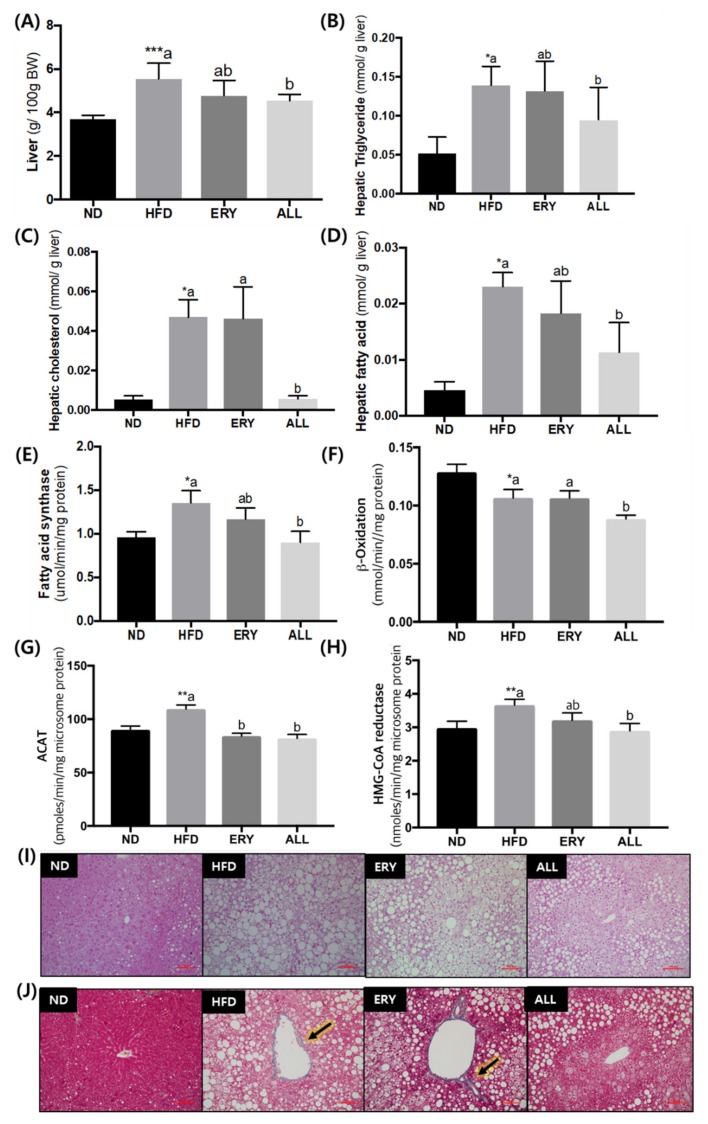
Effects of D-allulose supplementation for 16 weeks on; (**A**) Liver weight; (**B**–**D**) Hepatic lipid profiles; (**E**,**F**) Enzyme activities related to lipid metabolism; and (**I**) morphology. Data are mean ± SE; ND, normal diet (AIN-76); HFD, high-fat diet (AIN-76, 20% fat, 1% cholesterol); ALL, (HFD + 5% D-allulose). Mean values are significantly different for ND vs HFD, * *p* < 0.05, ** *p* < 0.01, *** *p* < 0.001; ^a, b^ Mean not sharing a common letter are significantly different among the groups at p < 0.05. (H) L: A ratio, leptin:adiponectin ratio; (I) Hematoxylin and eosin (H&E)-stained transverse section of epididymal fat and liver; Representative photomicrographs of the liver are shown at ×200 magnification; (J) Fibrillar collagens, primarily collagen I and III, are stained blue, as indicated by arrowheads; Representative photomicrographs of the liver are shown at ×200 magnification.

We measured the concentration of TG and TC in collected plasma from the tail vein every 4 weeks during the experiment, and these results are shown in Figure 2S–T. From the fourth week to the 12th week of the D-allulose supplement, plasma TG concentration was significantly decreased in the ALL group compared to the other HFD groups. From the fourth week of the D-allulose supplement to the end of the experiment, plasma TC concentration was significantly decreased in ALL group compared to HFD group. The lipid profiles with plasma obtained after a 24-hour fast at sacrifice was showed in Table 2. Total-C, HDL-C, nonHDL-C and apo A-I levels were significantly decreased in ALL group compared to HFD group. Also, the FFA, TG and apo B levels and HTR showed a decreasing tendency in the ALL group.

### 3.2. Suppression of Fatty Liver by D-Allulose Supplement in DIO Mice

The increased liver weight and hepatic lipid levels, including TG, fatty acids, and cholesterol, caused by HFD feeding, were significantly suppressed by ALL supplementation (Figure 2A). Moreover, the enzyme activities related to lipid metabolism, such as fatty acid synthase, β-oxidation, cholesterol acyltransferase, and 3-hydroxy-3-methylglutaryl-CoA reductase, were significantly decreased by D-allulose supplementation (Figure 2E–H). Consistent with these results, the ALL treatment reduced the accumulation of lipid droplets in the hepatic tissue (Figure 2I). MT staining of liver tissue revealed fibrotic stained a blue color in the HFD and ERY group, whereas it was absent in the ND and the ALL group. In particular, the ALL group showed a similar appearance to that of the ND group.

### 3.3. Effects of D-Allulose on SCFA Production in DIO Mice

**Figure 3:**
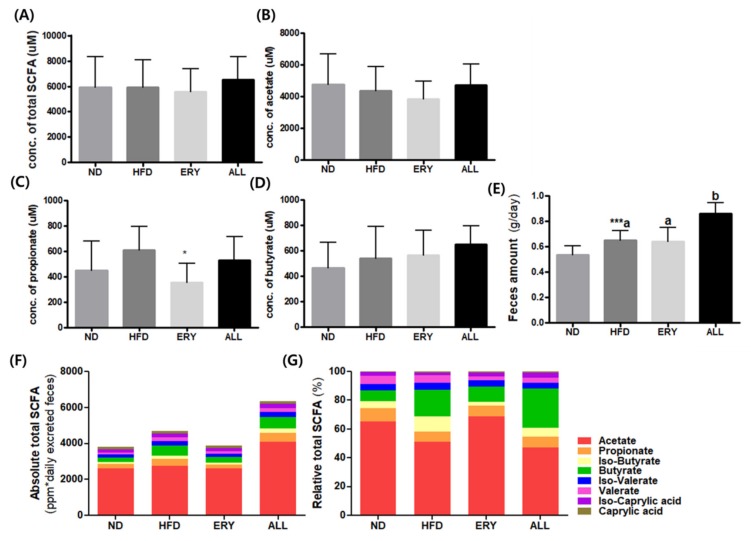
Effects of D-allulose on SCFA production. (**A**) Total SCFA production (**B**) Acetate; (**C**) Propionate; (**D**) Butyrate; (**E**) Daily fecal weight; (**F**) Absolute total SCFA adjusted with daily fecal weight (**G**) Relative total SCFA. Data are mean ± SE; ND, normal diet (AIN-76); HFD, high-fat diet (AIN-76, 20% fat, 1% cholesterol); ALL, (HFD + 5% D-allulose); SCFA, short-chain fatty acid.

The results of SCFA production are presented in Figure 3. Although the production of butyrate showed an increasing tendency in the ALL group, there was no significant difference in the SCFA production between the ALL and HFD groups. 

### 3.4. Effects of D-Allulose on Microbiome Modulation in DIO Mice

**Figure 4:**
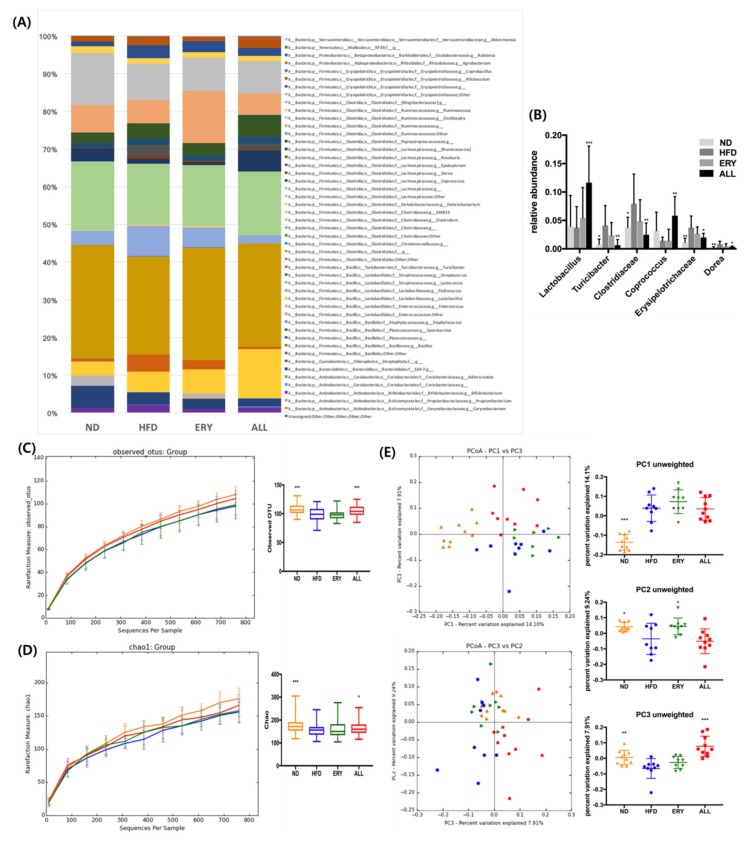
Effects of D-allulose on microbiota modulation. (**A**) Relative taxonomic abundance at the genus level. (**B**) Relative abundance of *Lactobacillus*, *Coprococcus*, *Coprobacillus*, *Turicibacter*, *Clostridiaceae*, *Dorea*, and *Erysipelotrichaceae*. (**C**) Observed operational taxonomic unit (OTU). (**D**) Chao 1. (**E**) Unweighted principal coordinates analysis and value of each principle coordinate dimension. Data are mean ± SE; ND, normal diet (AIN-76); HFD, high-fat diet (AIN-76, 20% fat, 1% cholesterol); ALL, (HFD + 5% D-allulose). Mean values are significantly different for HFD vs ALL, * *p* < 0.05, ** *p* < 0.01, *** *p* < 0.001.

**Figure 5:**
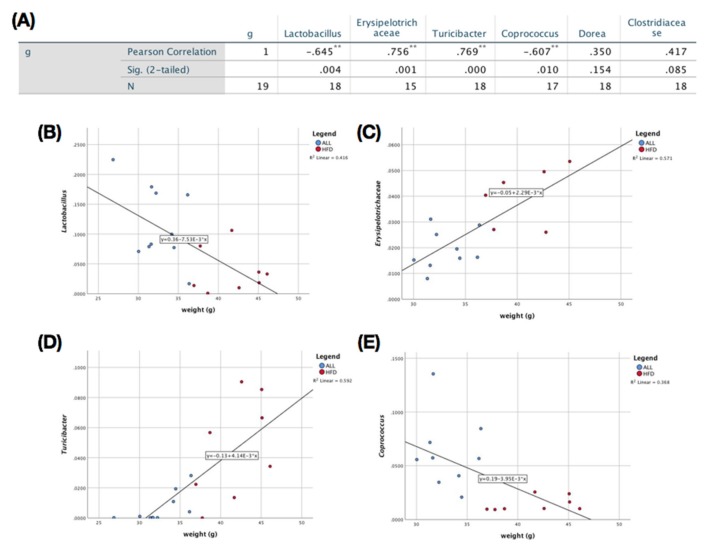
Correlation of microbiota with body weight. (**A**) Significance chart for Pearson’s correlation between microbiota and body weight. Correlation between body weight and the relative abundance of (**B**) *Lactobacillus*; (**C**) *Erysipelotrichaceae*; (**D**) *Turicibacter*, and (**E**) *Coprococcus*. HFD, high-fat diet (AIN-76, 20% fat, 1% cholesterol); ALL, (HFD + 5% D-allulose). Significance calculated through bivariate Pearson’s correlation analysis with body weight, * *p* < 0.05, ** *p* < 0.01.

The microbiome taxonomy result is shown in Figure 4A, B. At the genus level, there was a significant increase in *Lactobacillus*, *Coprococcus*, and *Coprobacillus*, in addition to a significant reduction in *Turicibacter*, *Clostridiaceae*, *Dorea*, and *Erysipelotrichaceae* in the ALL group compared with the HFD control (Figure 4A,B). Animals fed ALL and ND had a significantly higher alpha-diversity relative to HFD in both the Chao 1 (estimated OTU (operational taxonomic unit) richness and evenness) and observed OTU (diversity richness), as seen in Figure 4C,D. Both the ALL and the ND group had a significantly different beta-diversity from HFD, according to PC3 of the coordinate plot (Figure 4E). All of the significantly changed bacteria in the ALL group compared with the HFD group were checked for Pearson’s correlation with the body weight difference in the mice. Body weight had a significantly positive correlation with the *Turicibacter* and *Erysipelotrichaceae* genera (Figure 5C,D) and a significantly negative correlation with *Lactobacillus* and *Coprococcus* (Figure 5B,E). Changes in *Dorea* and *Clostridiaceae* did not show any significant correlation with body weight (Figure 5A).

## 4. Discussion

Previous studies have suggested that D-allulose can reduce body fat by regulating lipid metabolism [15,16]. In the same manner, the present study showed that D-allulose supplementation drastically decreased the body weight (Figure 1A,B) and body fat mass, without any change in food intake (Figure 1G,H)). D-allulose significantly increased FA oxidation and significantly decreased FA synthesis in eWAT (Figure 1J). To support these results, we measured the metabolic rate, which showed that VO_2_ and EE were significantly increased in the D-allulose group (Figure 1K–N). Our results are in accordance with part of a past study, in which D-allulose significantly increased the β-oxidation activity and its related mRNA expression (CPT1α, CPT2) [22]. The acyl CoA, the metabolite of β-oxidation, can used as a fuel for energy expenditure [27]. Taken together, D-allulose may increase the energy expenditure via the regulation of the mRNA expression and enzyme activity of β-oxidation in eWAT.

SCFAs are produced through the complex interactions of diet and the gut microbiome [28]. The interaction of SCFAs can regulate the host energy homeostasis and be evaluated as novel therapeutic targets for DIO [29]. According to previous studies, SCFAs has diverse roles in DIO, such as enhancing FFA oxidation and promoting beige adipogenesis and mitochondrial biogenesis. In particular, SCFAs led to significant increases in the expressions of G-protein coupled receptor (GPR)43 and GPR41 in the adipose tissue, which may further result in body weight reduction by enhancing TG hydrolysis and FA oxidation. In our study, the SCFAs production per 50 mg of feces had an increasing tendency in the ALL group compared to the HFD groups. However, as the D-allulose supplement significantly increased the daily fecal weight, the total absolute amount of SCFA production, when adjusted with daily fecal weight, can be more than that of other groups. Thus, the increased SCFA production by D-allulose supplement may result in FA oxidation, which is in accordance with increased enzyme activity in FA oxidation in the ALL group.

An increased richness in the gut’s microbial diversity has been negatively correlated with obesity and various disease states [30,31]. Furthermore, animal studies have indicated that treatment with probiotics or prebiotics can be a promising approach to alleviate these pathophysiological symptoms, by modulating the gut microbial ecology [28,29]. Firstly, we could observe the increase in the absolute total SCFA amount, a subset of key gut microbial metabolite, in the ALL group and the difference in relative total SCFAs among the groups in feces. Secondly, through the treatment of HFD feeding with D-allulose, there was an increase in both the alpha- and the beta-diversity in the gut microbiota when compared with the HFD control group (Figure 4E). Our study also found a significant increase in the relative abundance of *Lactobacillus*, which is known to improve gut barrier integrity, and *Coprococcus*, a known butyrate and propionate producer [32,33]. The abundance of both of these genera decreased with HFD-only consumption and increased in the D-allulose-consuming group compared to all the other groups (Figure 4A,B). Furthermore, some genera and families that were found to be at greater levels in obesity, such as *Dorea* and *Erysipelotrichaceae* [34,35], were significantly decreased in the D-allulose-fed animals relative to the HFD group. In addition, we observed a significant positive correlation between the abundance of *Erysipelotrichaceae* and body weight (Figure 5C).

Excessive cholesterol can build up in the arteries, which can lead to coronary heart disease and many other serious conditions such as stroke, insulin resistance and so on [1,36,37]. A high-cholesterol diet is a one of the predisposing factors for high cholesterol levels in blood [38]. The present study showed that HFD-fed mice increased the plasma and hepatic cholesterol and HMG-CoA reductase and ACAT activities compared to ND-fed mice;however, D-allulose supplement drastically decreased those values. Also, fecal cholesterol concentration was drastically increased by HFD with D-allulose. According to our previous study, D-allulose inhibits the dietary lipid absorption by the suppression of the CD36 expression, which is an important factor in the uptake of cholesterol and FA. In comparison of plasma TG levels with D-allulose, there was no significant difference in plasma collected after sacrifice; however, from the fourth week to the 12th week of the D-allulose supplement, the plasma TG concentration was significantly decreased in the ALL group compared to the other HFD groups. Although D-allulose could reduce the plasma TG levels, plasma TG concentration in HFD groups at the endpoint of the experiment was decreased overall, which may be due to various factors. In order to elucidate the exact mechanism of its action, an additional study is required for the correlation among D-allulose, HFD and TG metabolism. Our findings suggest that D-allulose ameliorated the HFD induced dyslipidemia by altering the lipid metabolism and increasing the excretion of fecal lipids.

High-fructose-fed animal studies have suggested that an increase in hepatic insulin resistance and increased glucose tolerance lead to non-alcoholic fatty liver disease (NAFLD) and the loss of tight junction proteins [39,40,41]. The loss of integrity of the tight junctions allows the leakage of endotoxins, induction of hepatic inflammation, and is associated with decreased levels of *Lactobacillus* and *Bacteroides* in the gut microbiota [40,41,42,43,44]. Several studies have also proved that the administration of *Lactobacillus* species protected the host against the onset of fructose-induced NAFLD [42,43,44]. In our study, the decrease in hepatic steatosis coincides with the increasing *Lactobacillus* population in the D-allulose-fed mice (Figure 2J, Figure 4B). It may have contributed to the amelioration of fatty liver disease and weight loss, by strengthening the epithelial barrier, inhibiting endotoxin translocation, and alleviating overall systemic and hepatic inflammation. Accordingly, a future experiment will be performed to find out whether the increasing *Lactobacillus* population associated with D-allulose supplementation can improve the inflammation.

## 5. Conclusions

In the present study, D-allulose supplementation was markedly effective in protecting the host against HFD-induced obesity and hepatic steatosis. It is plausible that these pathologies are mediated by the alteration of the gut microbiome profile and enhanced energy expenditure. Our findings suggest that D-allulose can exert its biological effects through modulating the gut microbiome.

## Figures and Tables

**Table 1:** Composition of experimental diets (% of diet, w/w).

Ingredient (g)	ND	HFD	ERY	ALL
Casein	200.00	200.00	200.00	200.00
D,L-Methionine	3.00	3.00	3.00	3.00
Corn Starch	150.00	111.00	111.00	111.00
Sucrose	500.00	370.00	320.00	320.00
Cellulose Powder	50.00	50.00	50.00	50.00
Corn Oil	50.00	30.00	30.00	30.00
Lard	-	170.00	170.00	170.00
Mineral Mix (AIN-76) ^1^	35.00	42.00	42.00	42.00
Vitamin Mix (AIN-76) ^2^	10.00	12.00	12.00	12.00
Choline Bitartrate	2.00	2.00	2.00	2.00
Cholesterol	-	10.00	10.00	10.00
*tert*-Butylhydroquinone	0.01	0.04	0.04	0.04
D-Allulose				50.00
Erythritol			50.00	
Total (g)	1000.0	1000.0	1000.0	1000.0
Calorie (kcal/kg)	3902	4584	4384	4384
Calorie (kcal/g)	3.902	4.584	4.384	4.384

ND, normal diet (AIN-76); HFD, high-fat diet (AIN-76, 20% fat, 1% cholesterol); ERY (HFD + 5% erythritol); ALL, (HFD + 5% D-allulose). ^1^ Mineral mix (AIN-76) (g/kg): calcium phosphate, 500; sodium chloride, 74; potassium citrate, 2220; potassium sulfate, 52; magnesium oxide, 24; manganous carbonate, 3.5; ferric citrate, 6; zinc carbonate, 1.6; cupric carbonate, 0.3; potassium iodate, 0.01; sodium selenite, 0.01; chromium potassium sulfate, 0.55; sucrose 118.03. ^2^ Vitamin mix (AIN-76) (g/kg): thiamin HCL, 0.6; riboflavin, 0.6; pyridoxine HCL, 0.7; nicotinic acid, 0.003; D-calcium pantothenate, 0.0016; folate, 0.2; D-biotin, 0.02; cyanocobalamin (vitamin B12), 0.001; retinyl palmitate premix, 0.8; DL-α-tocopheryl acetate, premix, 20; cholecalciferol (vitamin D3), 0.0025; menaquinone (vitamin K), 0.05; antioxidant, 0.01; sucrose, finely powdered, 972.8.

**Table 2:** Effect of D-allulose supplementations for 16 weeks on plasma lipid profiles in C57BL/6J mice fed a high-fat diet.

	ND	HFD	ERY	ALL
FFA (mmol/L)	0.20 ± 0.00	0.20 ± 0.00	0.19 ± 0.00	0.16 ± 0.00
TG (mg/dL)	0.86 ± 0.31	0.86 ± 0.09	0.93 ± 0.31	0.81 ± 0.16
Total-C (mmol/L)	3.56 ± 0.32	5.17 ± 1.21 ^***a^	4.48 ± 0.80 ^ab^	3.94 ± 0.32 ^b^
HDL-C (mmol/L)	0.95 ± 0.15	1.46 ± 0.34 ^***a^	1.15 ± 0.21 ^a^	0.88 ± 0.13 ^b^
Non-HDL-C (mmol/L)	2.61 ± 0.38	3.72 ± 0.93 ^***a^	3.33 ± 0.69 ^a^	2.65 ± 0.39 ^b^
ApoA-I (mg/dL)	31.52 ± 1.88	30.25 ± 0.90 ^a^	26.97 ± 1.18 ^ab^	25.53 ± 1.56 ^b^
ApoB (mg/dL)	6.03 ± 2.81	8.04 ± 2.92	6.51 ± 3.05	5.87 ± 2.64
ApoA-I/ApoB	6.54 ± 3.59	4.41 ± 2.18	5.02 ± 2.52	6.09 ± 4.71
HTR ^1^	25.75 ± 4.39	28.32 ± 3.58	25.92 ± 4.46	22.30 ± 2.48
AI ^2^	2.84 ± 0.72	2.58 ± 0.45	2.96 ± 0.69	3.54 ± 0.52

Data are mean ± SD. Significant differences between HFD vs ND are indicated; *** *p* < 0.001; ^a, b^ Mean not sharing a common letter are significantly different among the groups at p < 0.05. ND, normal diet (AIN-76); HFD, high-fat diet (AIN-76, 20% fat, 1% cholesterol); ALL, (HFD + 5% D-allulose). FFA, free fatty acid; TG, triglyceride; C, cholesterol; ApoA-I, apolipoprotein A-I; ApoB, apolipoprotein B; non-HDL-C = (Total-C) − (HDL-C); HTR ^1^, (HDL-C/Total-C) × 100; AI ^2^, atherogenic index = [(Total-C) − (HDL-C)]/HDL-C.

## Supplementary Materials

The following are available online at https://www.mdpi.com/2072-6643/12/2/352/s1.
