# Supplementary material for: Alteration of Microbiome Profile by D-Allulose in Amelioration of High-Fat-Diet-Induced Obesity in Mice
Source: Nutrients. 2020 Jan 29;12(2):352. doi: 10.3390/nu12020352 (PMC7071329; doi:10.3390/nu12020352)
Supplement: Supplementary file 1 [file nutrients-12-00352-s001.pdf]

Supplementary table 1. SYRCLE's tools risk of bias

Entry	Judgement	Support for judgement
Sequence generation	Low risk	Quote: "The mice were then randomly divided in to four group~."
Baseline characteristics	Low risk	Figure 1A, There was no significant difference in 0week bodyweight among the experiment groups
Allocation concealment	High risk	No allocation concealment
Random housing	Low risk	The animals were randomly housed during the experiment.
Blinding (performance bias)	High risk	We didn't blind
Random outcome assessment	Unclear	We didn't select animals for outcome assessment.<br>The result was analyzed using all animals (36mice).
Blinding (detection bias)	High risk	We didn't blind
In complete outcome data	Unclear	There was no incomplete outcome data
Selective outcome reporting	Low risk	There was no interference of selective outcome reporting.<br>We didn't select outcome data.
Other sources of bias	Low risk	
